# A specific, stable, and accessible LAMP assay targeting the HSP70 gene of *Trypanosoma cruzi*

**DOI:** 10.1128/spectrum.00172-25

**Published:** 2025-10-14

**Authors:** Sneider Alexander Gutierrez Guarnizo, Beth Jessy Condori, Luciana Basma, Shirley Equilia, Edith Malaga, Siena Defazio, Emily Arteaga, Jean Karla Velarde, Martín Obregón, Anshule Takyar, Carolina Duque, Jill Hakim, Freddy Tinajeros, Robert H. Gilman, Natalie Bowman, Monica R. Mugnier

**Affiliations:** 1 Department of International Health, Johns Hopkins University Bloomberg School of Public Health 25802, Baltimore, Maryland, USA; 2 Infectious Diseases Research Laboratory, Department of Cellular and Molecular Sciences, Universidad Peruana Cayetano Heredia 33216, Lima, Peru; 3 Universidad Catolica Boliviana San Pablo 28141 , Santa Cruz de la Sierra, Bolivia; 4 Asociación Benéfica Prisma (PRISMA), Lima, Peru; 5 Department of Pathology, Johns Hopkins School of Medicine, Baltimore, Maryland, USA; 6 W. Harry Feinstone Department of Molecular Microbiology and Immunology, Johns Hopkins Bloomberg School of Public Health, Baltimore, Maryland, USA; 7 Division of Infectious Diseases, School of Medicine, University of North Carolina at Chapel Hill, Chapel Hill, North Carolina, USA; Institut de Recherche pour le Developpement, Montpellier, France

**Keywords:** molecular diagnosis, LAMP-PCR, cost-effective method, HSP70 gene, acute chagas, congenital transmission

## Abstract

Diagnostic delays prevent most Chagas disease patients from receiving timely therapy during the acute phase when treatment is effective. Quantitative PCR (qPCR)-based diagnostic methods provide high sensitivity during this phase but require specialized equipment and complex protocols. More simple and cost-effective tools are urgently needed to expand early Chagas disease diagnosis in low-income endemic regions. Here, we present a loop-mediated isothermal amplification (LAMP) that targets a highly conserved region in the HSP70 gene of *Trypanosoma cruzi*, the causative agent of Chagas disease. This assay demonstrates species-specific amplification across multiple parasite genetic lineages while maintaining stability after 1 hour of incubation and at least 8 months of storage at −20°C. Moreover, the assay is at least 12 times less expensive than the TaqMan qPCR that is currently routinely used for acute Chagas diagnostics and is compatible with a low-cost CTAB-based DNA extraction method. Population-based validation in 100 infants born to Chagas-positive mothers in Santa Cruz, Bolivia, yielded a specificity of 100% and sensitivity exceeding 77% when compared to a TaqMan qPCR that targets satellite DNA. This cost-effective DNA extraction method and LAMP assay demonstrate potential for diagnosing Chagas disease in resource-limited endemic regions, particularly where qPCR is unavailable.

**IMPORTANCE:**

Chagas disease, caused by the parasite *Trypanosoma cruzi*, is a life-threatening illness that disproportionately affects resource-limited communities. Congenital Chagas disease, if diagnosed early, presents a unique opportunity for intervention, as treatment in newborns is highly effective with minimal side effects. However, early diagnosis is hindered by the high cost and limited availability of current molecular diagnostic methods in endemic regions. Our study introduces a simple, low-cost, and highly specific LAMP assay targeting the HSP70 gene of *T. cruzi*. This assay is user-friendly, stable under varying storage and incubation conditions, and designed for accessibility in underserved areas. By providing a detailed, open-access protocol and primers, we aim to facilitate the widespread adoption of this diagnostic assay, enabling earlier detection and treatment. This assay lays the groundwork for a new approach to Chagas disease management, potentially reducing the spread of Chagas disease and improving public health outcomes in vulnerable populations globally.

## INTRODUCTION

*Trypanosoma cruzi*, the causative agent of Chagas disease, is endemic to the Americas, primarily affecting vulnerable populations and perpetuating poverty (1). Annually, about 30,000 new cases are reported, with ~6 million people estimated to be infected (2). This devastating disease can cause severe clinical manifestations, including megaesophagus, megacolon, and dilated cardiomyopathy, leading to an estimated 12,000 deaths per year (2).

Chagas disease is transmitted through various routes, including vectorial (via triatomine bugs), oral, congenital, blood transfusion, organ transplantation, and lab accidents (3). Despite successful vector control and blood screening, congenital transmission remains a key source of new *T. cruzi* infections, accounting for approximately 22% of cases and serving as the primary source of new infections in non-endemic regions (4, 5). Once infected, patients enter an acute phase characterized by high parasitemia (6). During this phase, available treatments (benznidazole or nifurtimox) are highly effective and are well-tolerated in infants but can cause mild to severe side effects in adults (7, 8). The acute phase, especially in the context of congenital infection, offers a crucial window for intervention to prevent severe disease progression (7). Unfortunately, the absence of symptoms or the presence of non-specific ones often delays diagnosis. Consequently, it is estimated that fewer than 1% of people with Chagas disease receive treatment, largely due to delayed detection (9, 10).

Serological tests are routinely used to diagnose Chagas disease in the chronic phase, which is characterized by very low parasitemia (11). These tests, however, which rely on the presence of anti-*T*. *cruzi* antibodies, cannot reliably detect acute infections, including congenital infections, where anti-parasite antibody levels are typically low. Therefore, molecular diagnosis represents an ideal strategy for acute phase infection when parasitemia is high and infection is treatable (12–16). The most common molecular diagnostics for acute phase infection are quantitative PCRs (qPCRs) targeting nuclear satellite DNA (SatDNA) (13, 17, 18), but these are expensive and require sophisticated equipment that is often unavailable in the low- and middle-income regions affected by Chagas disease (19, 20). This highlights the urgent need for cost-effective, robust, and simple DNA-based diagnostic assays. Without such tools, the timely diagnosis of congenital Chagas and other acute forms of the disease will remain challenging, leaving infections untreated and increasing the risk of irreversible chronic Chagas complications (21, 22).

To help address the limitations of existing molecular diagnostics, two major isothermal DNA amplification technologies have emerged as alternatives to standard qPCR: loop-mediated isothermal amplification (LAMP), introduced in 2000 (23), and recombinase polymerase amplification (RPA), introduced in 2006 (24). Though it utilizes a more complex primer design, LAMP is notably less expensive than RPA (25) and has been widely used in diagnosing other infectious diseases such as COVID-19 and malaria, demonstrating its potential as a tool for population level diagnosis (26–28). Additionally, compared to qPCR, LAMP requires less hardware and is more tolerant of amplification inhibitors (29).

Although LAMP assays have been developed for Chagas diagnosis previously (30–33), existing assays have shown some limitations, including false positives (30), inability to discriminate between infection with *T. cruzi* and infection with other trypanosomatids (31, 34), and restricted access to protocols (30, 32). Additionally, there is some evidence that the extreme genetic variability of *T. cruzi* strains might affect LAMP assay performance (35). The limitations of existing LAMP assays highlight the need for alternative targets.

A promising alternative is to target the conserved protein-coding genes of *T. cruzi*, which have yet to be explored in LAMP assays for Chagas disease. Protein-coding genes are particularly appealing because of their greater genetic stability, a critical factor for reliable primer alignment (36). While often overlooked in diagnostics due to their low copy numbers, protein-coding genes in *T. cruzi* often exist as multigene families, affording both the high copy numbers required for test sensitivity as well as the sequence conservation needed to develop a sufficiently specific diagnostic.

In this study, we developed a novel LAMP assay targeting a highly conserved region in the multicopy, protein-coding *HSP70* gene family of *T. cruzi*. Our LAMP protocol successfully amplified *T. cruzi* DNA from five distinct parasite genetic lineages without amplifying DNA from the related parasites *Trypanosoma rangeli* and *Leishmania* spp. The assay proved robust and stable under various conditions, including a variety of incubation times, medium-term storage at +4°C, and long-term storage at −20°C. It also produced consistent results across various sample types. Evaluating congenital Chagas in a population of Santa Cruz, Bolivia, the assay showed optimal specificity and high sensitivity. This portable method could enable large-scale diagnosis and patient monitoring in endemic regions lacking economic or infrastructural resources.

## RESULTS

**Fig 1:**
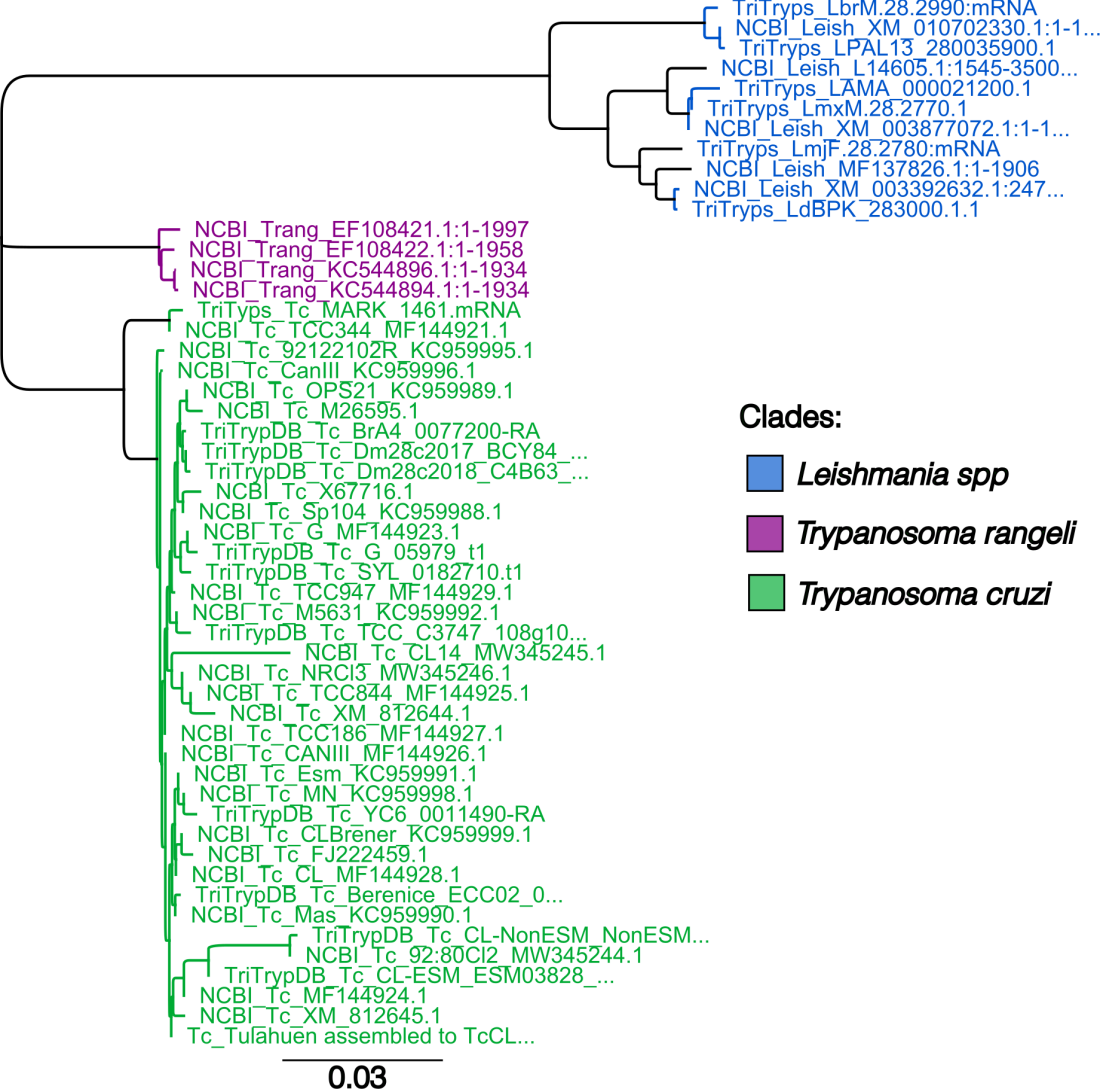
The *HSP70* sequence is a molecular marker that differentiates *T. cruzi* from closely related trypanosomatids. Neighbor-joining tree analysis based on *HSP70* sequences discriminates *Leishmania* spp. (blue), *T. rageli* (purple), and *T. cruzi* (green). The horizontal scale represents the evolutionary distance. A scale of 0.03 means an average of three substitutions per 100 nucleotides.

The *T. cruzi HSP70* subfamily genes, which serve as molecular chaperones involved in protein folding and transport, are an appealing target for a LAMP assay. In *T. cruzi*, there are an average of 88 *HSP70* genes per genome (TriTryp DB, Release 68). Moreover, the gene appears to be highly species-specific, in terms of both organization and sequence, potentially allowing for the specific discrimination of *T. cruzi* from other trypanosomatid parasites (37, 38). To assess the potential of *T. cruzi HSP70* as a diagnostic target, we performed a neighbor-joining tree analysis to compare the *HSP70* sequences across various *T. cruzi* strains (*n* = 36) and two closely related trypanosomatids, *Leishmania* spp. (*n* = 11), and *T. rangeli* (*n* = 4), which infect humans in the same geographic region as *T. cruzi*. The *T. cruzi HSP70* sequences cluster in a distinct clade compared to the *HSP70* of *T. rangeli* and *Leishmania* spp. (Fig. 1; File S1). The *T. cruzi HSP70* sequences exhibited a high identity of 98.63% (File S1), indicating that this sequence is highly conserved and serves as a feasible target for designing LAMP primers.

**Fig 2:**
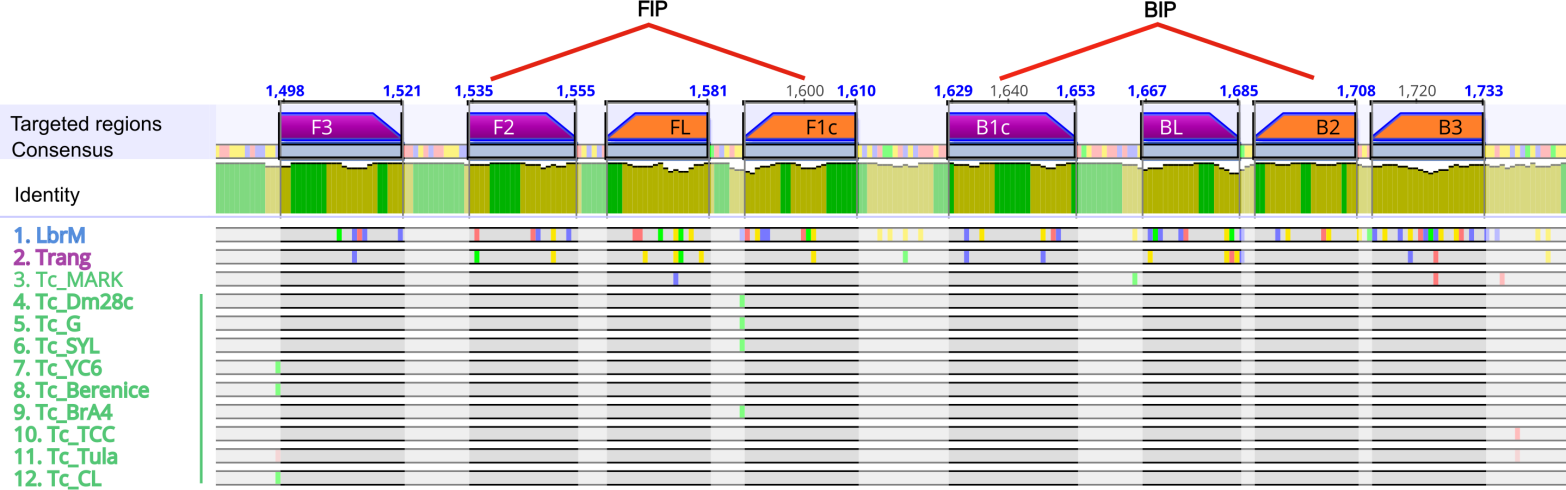
Primers for LAMP*_TcHSP70_* align to regions that distinguish *T. cruzi* from *Leishmania* and *T. rangeli*. The HSP70 sequences from 12 representative strains of *Leishmania*, *T. rangeli*, and *T. cruzi* were aligned using the Clustal Omega algorithm. The regions where the primers align in forward and reverse are shown as purple and orange bars, respectively. The inner primers (FIP and BIP) align to two distinct regions, connected by red lines. Nucleotide variants are depicted as small colored bars over the sequences represented as horizontal gray bars. All *T. cruzi* sequences, except *T. cruzi* marinkellei, which does not infect humans, show 100% identity in the primer-targeted regions. The figure was generated using Geneious Prime software. Sequences named on the left: (1) *L. braziliensis*, (2) *T. rangeli*, (3) *T. cruzi* marinkellei, (4) *T. cruzi* Dm28c, (5) *T. cruzi* G, (6) *T. cruzi* Sylvio, (7) *T. cruzi* YC6, (8) *T. cruzi* Berenice, (9) *T. cruzi* Brasil A4, (10) *T. cruzi* TCC, (11) *T. cruzi* Tulahuen, and (12) *T. cruzi* CL.

Next, the LAMP*_TcHSP70_* primers were designed along a region of 236 bp at the end of the *HSP70* open reading frame. This region was conserved across *T. cruzi* strains but included sequence variations in *Leishmania* spp. and *T. rangeli* to minimize primer binding to these other species. Eight subregions were defined (F3, F2, FLc, F1, B1c, BL, B2c, and B3c), resulting in six primers, the forward outer primer (F3), the backward outer primer (B3), the forward loop primer (FL), the backward loop primer (BL), the forward inner primer (FIP), and the backward inner primer (BIP). The FIP and BIP primers create the forward and backward loop, respectively, and align in two regions each. FIP aligns in F2 and F1c, while BIP aligns in B2c and B1c (Fig. 2; File S2 and S3). The designed primers showed Gibbs free energy (ΔG) values between −3.61 and −11.49, with primers F3 and F1c showing moderate risk for self and heterodimers (File S4).

**Fig 3:**
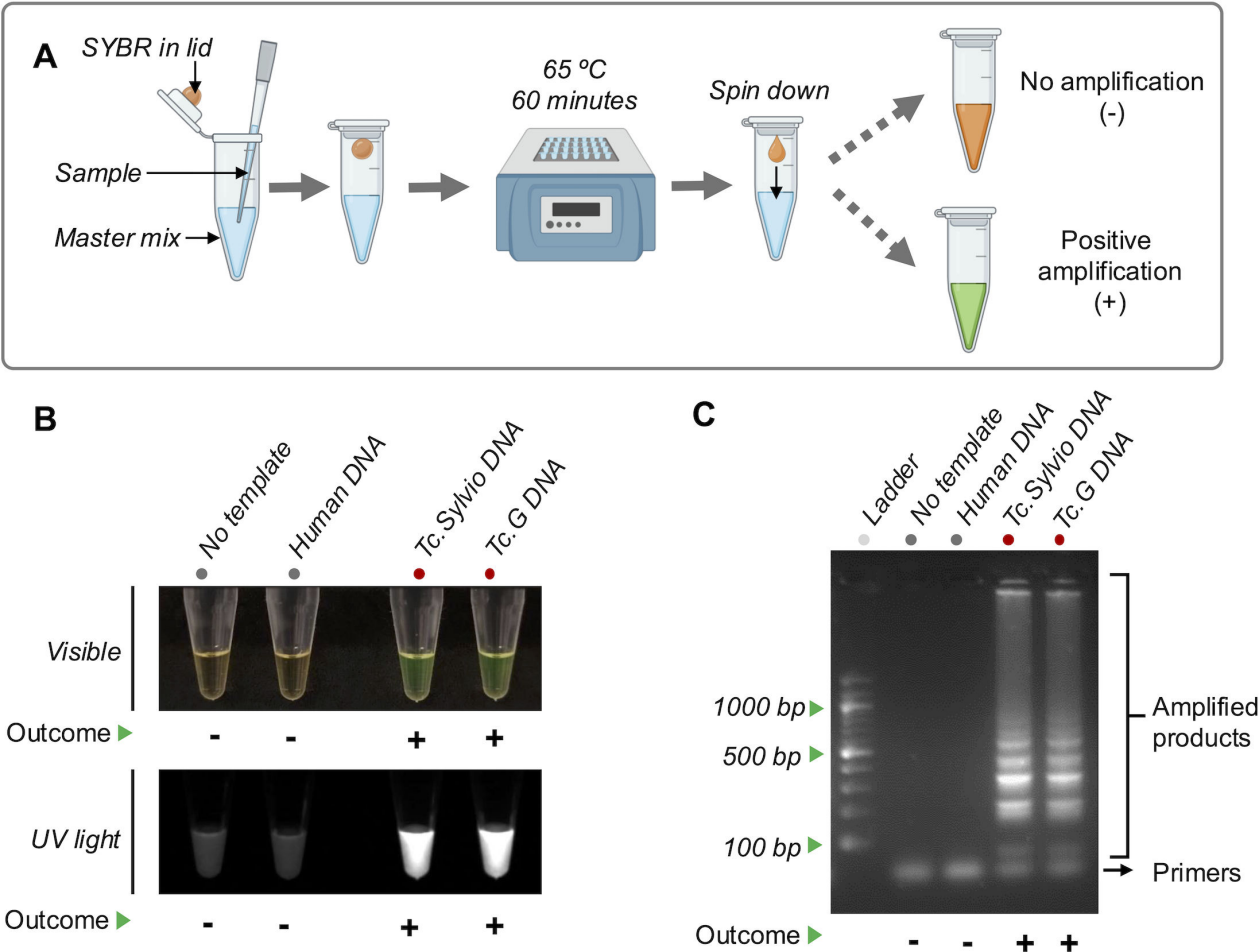
LAMP*_TcHSP70_* amplifies *T. cruzi* DNA. (**A**) Workflow for LAMP*_TcHSP70_* amplification. (**B**) Visualization of the amplified products by visible light and UV light. 1 ng DNA was loaded per sample. (**C**) Visualization of the amplified products by electrophoresis. Representative agarose gel of three replicates showing the classic pattern of LAMP product. Three microliters of the amplified products was visualized on a 2% agarose gel pre-stained with 0.2 µg/mL ethidium bromide. A 100 bp DNA ladder was used as a reference for determining Amplicon sizes. Created in BioRender (2025). https://BioRender.com/8h9gegb*.*

To test the LAMP*_TcHSP70_* primers, we conducted a preliminary reaction evaluating the amplification under the conditions established by Ordoñez et al.(33). This involved incubating for 60 minutes at 65°C and adapting it to the SYBR Green-based one-pot system (39) (Fig. 3A). We evaluated two representative *T. cruzi* strains (Sylvio and G) and two negative controls (no template control and human DNA control). A positive result was visible as a color change from orange to green under visible light or as green fluorescence under UV light (Fig. 3B). The results were confirmed by agarose gel electrophoresis, which showed amplification bands for both *T. cruzi* strains and no bands for the negative controls. Additionally, the agarose gel did not show significant formation of primer dimers (Fig. 3C).

**Fig 4:**
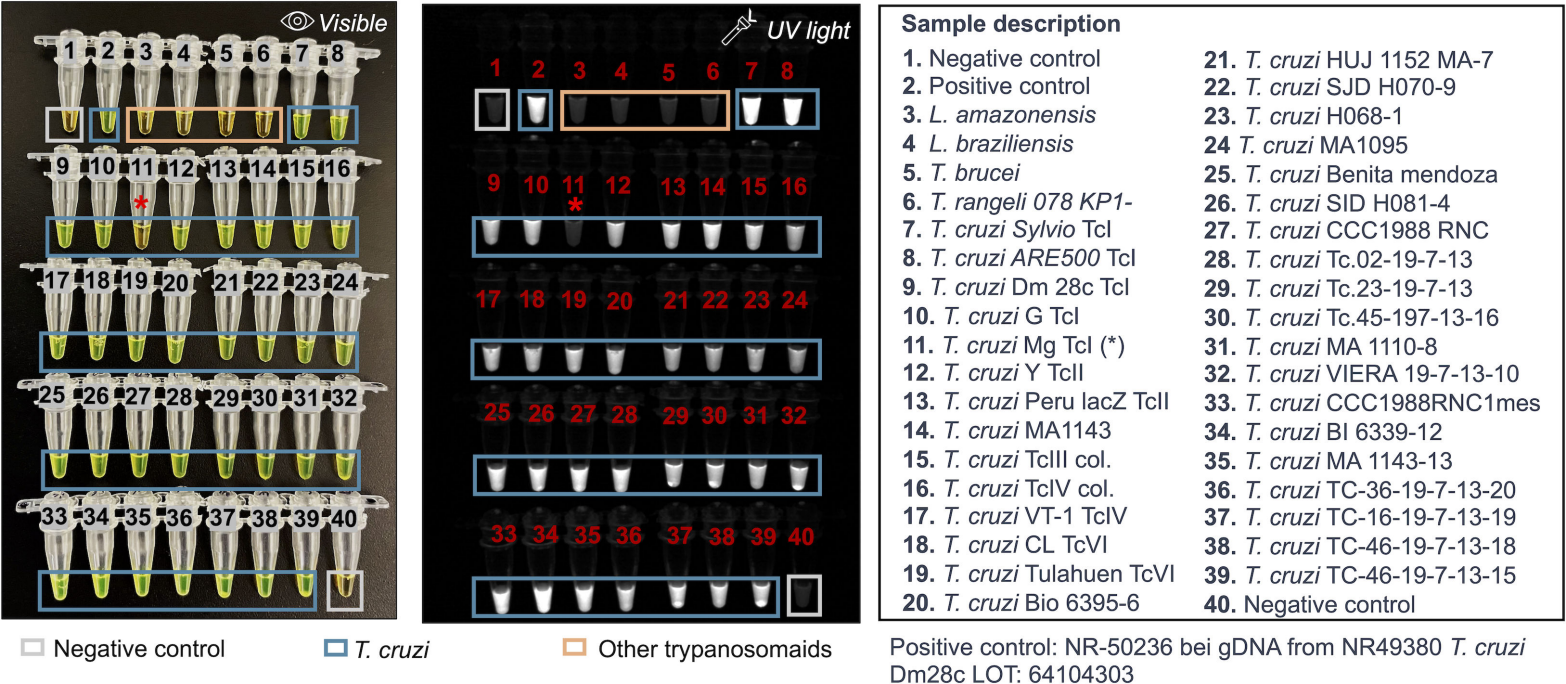
LAMP*_TcHSP70_* shows high specificity and sensitivity using parasite-derived DNA. Forty samples were run in parallel. representative captures of two replicates evaluated by visible and UV light. For all samples, 1 ng of purified DNA was seeded.

Next, we evaluated the specificity of the LAMP*_TcHSP70_* assay for *T. cruzi* and its ability to amplify various distinct strains. We tested 40 samples, including two negative controls, two *Leishmania* species, *T. brucei*, *T. rangeli*, and 34 *T. cruzi* strains. The LAMP*_TcHSP70_* did not amplify DNA from any species other than *T. cruzi*. The LAMP*_TcHSP70_* showed a preliminary sensitivity of 97% (95% confidence interval [CI]: 85% to 100%), and a specificity of 100% (95% CI:40% to 100%). Only the isolate *T. cruzi* Mg TcI from Colombia did not show positive amplification (Fig. 4). These results may be influenced by several factors, including sample integrity, the presence of inhibitors, and potential genomic sequence variation.

**Fig 5:**
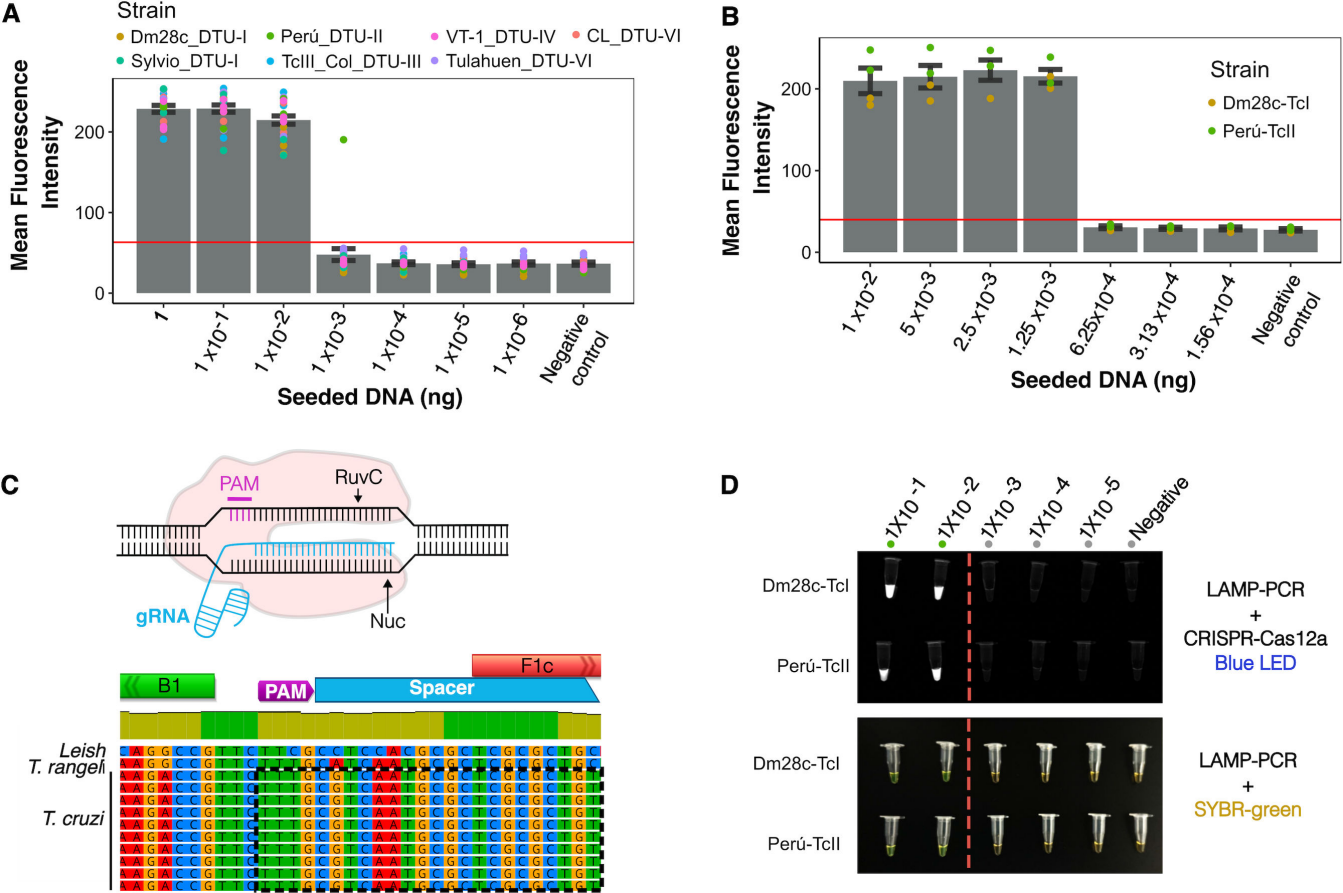
The LAMP*_TcHSP70_* assay shows a consistent LoD across multiple strains and DTUs, with a LoD in the order of picograms. (**A**) LoD after the first round of serial DNA dilutions using a factor of 10. (**B**) LoD after the first round of serial DNA dilutions using a factor of 2. (**C**) Design of CRISPR-Cas12a system. (**D**) LoD after using LAMP*_TcHSP70_* coupled to CRIPRcas12a system. Three independent replicates per strain. Mean fluorescence intensity was estimated using the ROI manager tool of fiji two software. (**A and B**) Error bars indicate standard error. The horizontal red line highlights the mean fluorescence intensity reached by the negative control.

After confirming that the LAMP*_TcHSP70_* has good specificity and promising sensitivity, we assessed the analytical limit of detection (LoD). To achieve this, we serially diluted *T. cruzi* DNA to identify the lowest DNA amount yielding a positive amplification. We evaluated seven reference strains representing the five different *T. cruzi* lineages known as discrete typing units (DTUs). DTUs broadly represent the parasite’s genomic variability. Initial 10-fold serial dilutions established a preliminary LoD of 0.01 ng (Fig. 5A and B). To refine this estimate, we conducted further twofold dilutions for two representative strains, resulting in a final LoD of 1.25 pg. The LoD was consistent across different *T. cruzi* lineages and is equivalent to 0.068 ± 0.032 parasites (Fig. 5C and D; File S5). When comparing the LAMP*_TcHSP70_* to the routine TaqMan SatDNA qPCR used for acute Chagas diagnosis (40), the LAMP*_TcHSP70_* was able to detect DNA in samples with a SatDNA qPCR Ct value up to 28.37 ± 0.26 (File S6). These results suggest that LAMP*_TcHSP70_* might have optimal sensitivity for detecting acute Chagas infections characterized by high parasitemia.

We next evaluated whether the addition of CRISPR-Cas12a to the LAMP*_TcHSP70_* could improve the LoD as demonstrated in previous studies targeting different pathogens (39, 41–44). We designed a gRNA to specifically recognize a sequence between the B1 and F1c regions, where the inner primers align to form the loops (Fig. 5C). As shown in Fig. 5D, although the CRISPR-Cas12a system improved the discrimination between positive and negative amplification, it did not significantly extend the LoD. Our cost analysis revealed that the standard TaqMan SatDNA PCR is 14.3 times more expensive than LAMP with SYBR Green and 12.9 times more expensive than LAMP with the Cas12a system, highlighting the cost-effectiveness of the LAMP approach (File S7).

**Fig 6:**
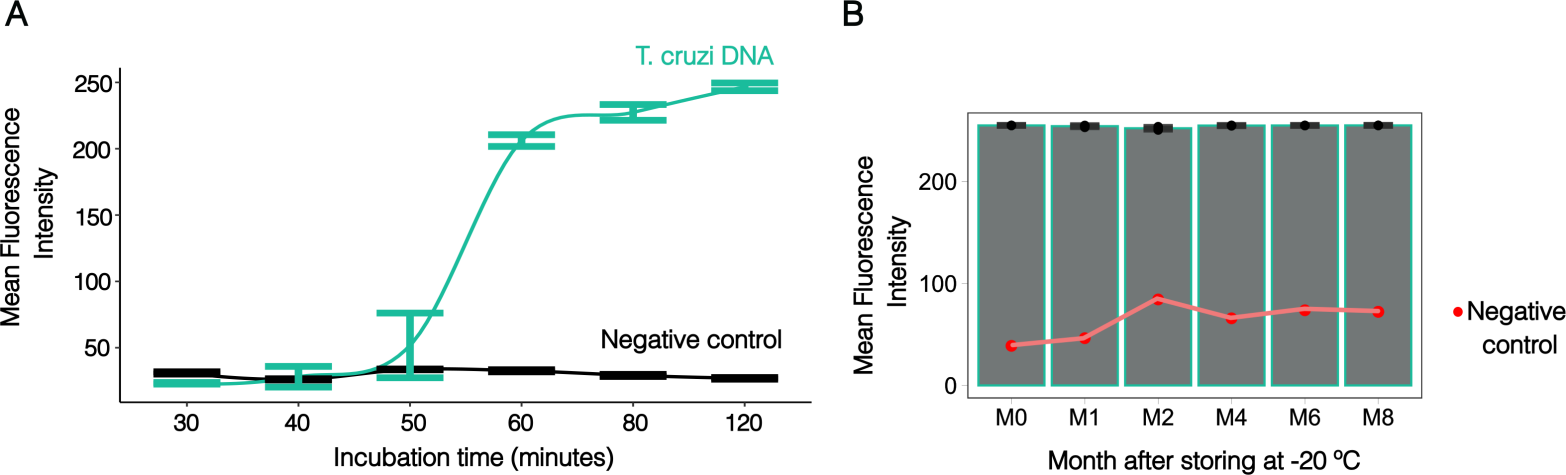
The LAMP targeting *T. cruzi HSP70* remains stable even after extended incubation and storage times. (**A**) Extended incubation times do not reveal non-specific amplification in negative controls. Data represent four technical replicates per incubation period. Human DNA (negative control) was seeded at 50 ng, while *T. cruzi* DNA was seeded at 2 pg. (**B**) Master mix storing for 8 months does not affect LAMP performance. Data represent three technical replicates per storing period. One reaction was used for negative control. Human DNA (negative control) was seeded at 50 ng, while *T. cruzi* DNA was seeded at 1 ng. Error bars indicate standard error. Mean fluorescence intensity was estimated by ROI area using Fiji software.

Once the LoD for our system was established, we then assessed the minimum incubation time required for optimal amplification of 2 pg of DNA of the template. Our findings demonstrate that incubation at 65°C for 60 minutes is needed to detect low-abundance targets (Fig. 6A; File S9). Although our preliminary analysis did not reveal nonspecific amplification after 120 minutes of incubation (Fig. 6A; File S9), we further evaluated this by including a set of 24 reactions using only human DNA. These reactions revealed nonspecific products at 2 hours, but stable results were still observed at 60 minutes (Fig. S1). When storing the complete master mix at 4°C, we found it remained stable for up to 4 weeks, with a significant decline in signal intensity observed after 5 weeks, indicating a loss of performance (Fig. S2). We then evaluated the long-term stability of the complete master mix at −20°C. After 8 months, the master mix showed no significant difference in mean fluorescence intensity (Fig. 6B; File S8). These results indicate that LAMP*_TcHSP70_* is stable for prolonged incubation and storage at −20°C. The reaction can thus be prepared in large batches to streamline daily sample processing.

**Fig 7:**
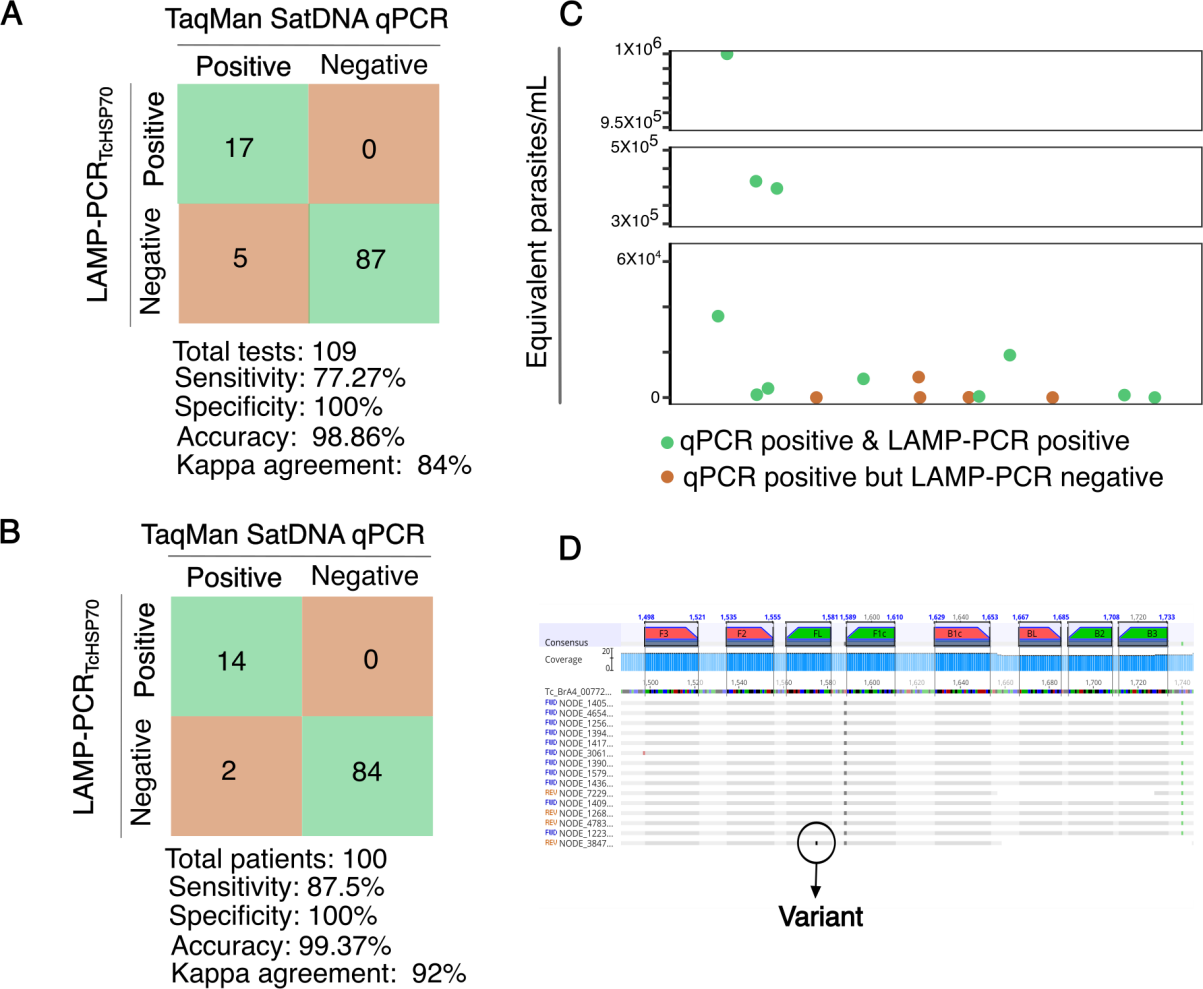
The LAMP*_TcHsp70_* assay exhibits high specificity and accuracy but lower sensitivity compared to SatDNA-qPCR. (**A**) Sensitivity, specificity, and accuracy per test. (**B**) Sensitivity, specificity, and accuracy per patient diagnosis. (**C**) Positive and negative LAMP*_TcHSP70_* results in relation to parasitic load (parasites/mL) as estimated by the qPCR calibration curve. (**D**) Alignment of *HSP70* sequences from clinical isolates.

Having demonstrated good specificity, sensitivity, and stability of our LAMP*_TcHSP70_* assay with parasite isolates, we then evaluated its performance with clinical samples. A total of 100 infants born to Chagas-positive mothers were analyzed; for each infant, DNA was extracted from peripheral and/or umbilical cord blood. Ninety-two infants had a single sample tested, while eight infants (infant IDs: 1, 2, 15, 19, 27, 31, 34, and 76) had more than one sample evaluated, resulting in a total of 109 samples (File S9). We compared both the sample-based and patient-based performance of LAMP*_TcHSP70_* with the TaqMan-based SatDNA-qPCR, the routine diagnostic test for congenital Chagas disease, using a blinded approach. For the 109 samples evaluated, LAMP demonstrated a sensitivity of 77.27% (95% CI: 54.63% to 92.18%) identifying 17 out of 22 positive samples by qPCR, a specificity of 100% (95% CI: 95.85% to 100.00%), an accuracy of 98.86% (94.63% to 99.95%), and a Kappa agreement of 84% (95% CI: 71.12% to 97.76%) (Fig. 7A). When analyzing results per patient, where at least one positive sample classified the infant as positive, LAMP showed a sensitivity of 87.5% (95% CI: 61.65% to 98.45%) identifying 14 out of 16 positives by qPCR, a specificity of 100% (95% CI: 95.7% to 100.00%), an accuracy of 99.37% (95% CI: 95.2% to 100%), and a Kappa agreement of 92% (95% CI: 81.41% to 100%)(Fig. 7B). Notably, LAMP*_TcHSP70_* performed better with samples with high parasitemia but also detected samples with low parasitemia, as shown in Fig. 7C. These findings suggest that LAMP*_TcHSP70_* is a viable alternative to SatDNA-qPCR for diagnosing acute Chagas, especially in resource-limited settings.

Besides sensitivity and variability in sample quality, the failure of LAMP to amplify certain samples may also stem from issues with primer alignment. *HSP70* is a highly conserved gene, but *T. cruzi* parasites exhibit significant genomic plasticity (45). The inability of our LAMP to amplify certain samples detected by standard PCR (e.g., infant IDs: 4, 19) might also be the result of genomic variations, such as mutations or low gene dose, that affect LAMP performance. Additional DNA from the patients tested was unavailable to investigate *HSP70* sequence variations potentially impacting primer alignment, so we analyzed the *HSP70* sequence of 15 clinical *T. cruzi* isolates from additional patients from the same population in Santa Cruz, Bolivia. The analysis confirmed that the *HSP70* sequence is generally highly conserved, revealing only one variant (p1575 T - > G). This variant does not affect the 3′ or 5′ primer ends, suggesting a likely low impact on primer performance (Fig. 7D). However, these findings underscore that local parasite populations may harbor low-frequency variants that could impact primer alignment for the LAMP and other assays.

Finally, to enable LAMP-PCR implementation in low-resource settings, we evaluated the compatibility of a cost-effective DNA extraction method with the LAMP-PCR assay. DNA extracted using a cetyltrimethylammonium bromide (CTAB) buffer-based protocol was compared to the reference commercial kit (Roche High Pure PCR Template Preparation Kit). The CTAB-based method takes about 50 minutes to complete, and non-significant differences were observed in DNA yield or LAMP-PCR performance between the two methods (Fig. S3). After a cost analysis, the CTAB-based method was at least four times cheaper than the previously mentioned commercial kit (File S7). These results indicate that the CTAB-based DNA extraction method can be considered as a viable alternative for DNA extraction in LAMP-PCR protocols targeting blood-borne pathogens such as *T. cruzi*.

## DISCUSSION

Early diagnosis is critical for the treatment of Chagas disease, but is hampered by the high cost of traditional methods. Here, we present a new specific, stable, and accessible LAMP assay based on the detection of *T. cruzi HSP70* gene.

LAMP has emerged as a cost-effective method for expanding the use of molecular diagnostics at the population level (25), yet only a few assays exist for detecting *T. cruzi*. Primarily focused on established qPCR targets, these assays show variable sensitivity (69.2%–97%) (30–32) and frequently suffer from non-specific amplification, resulting in false positives (30) or an inability to distinguish *T. cruzi* infection from infection with related trypanosomatids (31, 34), leading to a reduced specificity. Moreover, the primer sequences for one of the most promising SatDNA-targeting LAMP have not been made publicly available, limiting its use and independent validation (30, 32, 46). Given these limitations, we sought to develop an open-access LAMP assay targeting a novel, highly specific *T. cruzi* sequence.

Our phylogenetic analysis confirmed the effectiveness of *HSP70* in distinguishing *T. cruzi* from related species, enabling the design of LAMP primers targeting eight highly conserved regions. Using this primer set, we found that our LAMP*_TcHSP70_* assay demonstrated 100% specificity when tested with reference strains and clinical samples, with no false positives even after 1 hour of incubation time. This indicates a lack of non-specific primer interactions, a common issue in LAMP assays exceeding 30 minutes of incubation (47, 48). Furthermore, unlike 18S rRNA targeting assays, our HSP70-based assay avoids cross-amplification with *Leishmania*, further highlighting its high specificity. Additionally, we showed that the LAMP*_TcHSP70_* master mix is highly stable during long-term freezing and shorter-term storage 4°C, facilitating large batch preparations and streamlining master mix preparation. Future experiments with very low-input DNA will be valuable to refine the impact of master mix storage on assay performance.

Despite having a lower copy number compared to SatDNA, kDNA, or 18S rRNA, the *HSP70* targeted by our LAMP demonstrates high analytical sensitivity with a LoD equivalent to less than 0.07 parasites per reaction. This sensitivity is reasonable for diagnosing acute Chagas, especially congenital Chagas, which is characterized by high parasitemia, particularly after the first month of birth. When analyzing clinical samples, the LAMP*_TcHsp70_* showed a sensitivity of 87.5%. This somewhat reduced sensitivity relative to existing qPCR assays (0.01–0.1 parasite/mL of blood) is likely at least in part the result of differences in target copy number: while parasites harbor an average of 88 copies of *HSP70* per genome, they harbor 10^4^ to 10^5^ copies of the DNA satellite region (49). Notably, other LAMP-PCR assays targeting the high-copy satellite DNA show variable concordance with qPCR. Besuschio et al. (32) reported 92% agreement, while more recently, Rojas et al. (30) found 77%–88%. Despite targeting a lower-copy gene, our LAMP-_TcHSP70_ assay shows an agreement of 84% and thus promising performance for diagnosing acute Chagas disease when qPCR is not available.

Although we cannot rule out the fact that mutations affected the assay performance, our evidence suggests *HSP70* mutations are unlikely to be the cause of false-negative results. Preliminary sequencing of a subset of clinical isolates from Santa Cruz, Bolivia, did not reveal any mutations in the parasites circulating locally that would interfere with primer amplification. *T. cruzi* reproduces asexually, primarily through binary fission, so we would not anticipate rapid accumulation of mutations in this gene under normal conditions. Nevertheless, our understanding of the genetic diversity of *T. cruzi* in the clinical setting is limited. Additional genome sequencing from clinical samples tested with the LAMP-PCR assay is needed to ensure the optimal design of LAMP primers and to clarify whether gene variants or copy number variations in the *HSP70* gene affect the sensitivity of the LAMP-_TcHSP70_ assay

Another explanation for the lower sensitivity of LAMP*_TcHsp70_* when compared to qPCR is the possibility that some samples contained LAMP inhibitors. Notably, in this study, we used the Bst 2.0 polymerase, a reliable enzyme for DNA amplification in LAMP assays. The advent of more advanced polymerases, which amplify not only DNA but also RNA templates, could significantly enhance both the sensitivity and reaction speed of the assay.

Because our assay failed to detect *T. cruzi* DNA in 2/16 infants, we explored the integration of a CRISPR-Cas12a system to improve the assay’s sensitivity. Though it did not improve sensitivity, CRISPR-Cas12a did improve the signal-to-noise ratio of the assay, with a more refined readout compared to SYBR Green, which interacts non-specifically with any DNA in the reaction. Thus, the incorporation of the Cas12a system could be valuable in circumstances where samples are suffering from high background amplification.

Regardless of the detection method (SYBR Green or Cas12a), our LAMP*_TcHSP70_* remains at least 12 times cheaper than traditional qPCR assays. Complementarily, using a CTAB buffer-based extraction method, it was possible to isolate DNA directly from whole blood without compromising LAMP-PCR performance at ¼ of the cost. This streamlined workflow enabled both DNA extraction and LAMP-PCR to be completed in under 2 hours. Moreover, we have made the entire LAMP*_TcHSP70_* and CTAB-based protocols publicly available (50, 51). The assay’s ease of use, low cost, and open protocol not only facilitate independent validation of the method but also provide a promising option for large-scale, low-cost diagnosis of suspected acute Chagas infection.

Such large-scale diagnosis is particularly critical in the context of congenital Chagas, an especially important avenue for control of Chagas disease. Approximately 22% of new cases are congenitally transmitted. As a form of acute infection, congenital Chagas is uniquely treatable (5). When diagnosed and treated within the first year of life, congenital *T. cruzi* infection has a >90% cure rate (4). Therefore, large-scale diagnosis of newborns in endemic regions could significantly increase the number of detected and thus treated cases of acute Chagas. Such population-level diagnosis has been infeasible until now, for reasons related to both logistics and cost. LAMP*_TcHSP70_* overcomes these challenges, being stable and specific and exhibiting good sensitivity when applied to clinical samples.

This study highlights a new target for detecting acute Chagas disease and reinforces LAMP as a promising, low-cost tool to expand diagnosis and treatment in low-income regions. Although not yet a replacement for standard qPCR, LAMP-_TcHSP70_ offers a valuable alternative where qPCR is unavailable and stands to benefit from continued optimization of enzymes, primers, and reagents.

## MATERIALS AND METHODS

### DNA and strains of reference

We used DNA from reference strains obtained from american type culture collection (ATCC) and biodefense and emerging infections research resources repository (BEI) resources, including *T. cruzi* strains representative of DTU I (Dm28c NR-49380, Sylvio-X10 ATCC-50823, G NR-49382), DTU II (Y NR-46429, Peru LacZ Clone 4 NR-18960), DTU IV (TcVT-1 NR-46428), and DTU VI (Tulahuen LacZ clone C4 NR-18959, CL). Additionally, we used DNA from *Leishmania amazonensis* (NR-49247) and *Leishmania braziliensis* (NR-50608). DNA from *T. cruzi* Mg Tc I, *T. cruzi* TcIII col, and *T. cruzi* TcIV col strains was provided by Dr. Juan David Ramirez from Universidad del Rosario. DNA from the reference strain *T. rangeli* 078 KP1(-) was donated by Dr. Daniel Urrea from the LIPT laboratory at Universidad del Tolima. All remaining isolates were obtained from Dr. Gilman’s repository (IRB00007176).

### Population of study

Sample collection was approved by the Institutional Review Boards of the University of North Carolina at Chapel Hill (IRB 19-3014) and Hospital De La Mujer Dr. Percy Boland (Protocol 036). Pregnant women were recruited at Hospital De La Mujer Dr. Percy Boland in Santa Cruz, Bolivia, over 12 months between June 2023 and June 2024. A total of 1,344 mothers were enrolled, 252 of whom were seropositive for Chagas disease. Umbilical cord blood samples were collected at birth, and infant peripheral blood was obtained post-delivery. Based on routine TaqMan SatDNA qPCR, 16 infants were diagnosed with congenital Chagas. This study evaluated samples from 100 infants, including 84 controls and 16 cases of congenital Chagas that had sufficient DNA left over after performing the routine diagnostics. All samples/patient IDs were deidentified and not known to anyone outside the research group.

### Commercial kit DNA extractions

DNA from parasite pellets was purified using the QIAamp DNA Blood Mini Kit (Catalog number or CN: 51104) according to the manufacturer’s instructions. DNA from patient samples was purified using the High Pure PCR Template Preparation Kit (Roche Diagnostics GmbH, Mannheim, Germany; REF: 11796828001) as previously described (40). An internal amplification control (IAC) was prepared at 40 pg/µL. For blood samples, 300 µL of blood was mixed with Guanidine/EDTA and 5 µL of IAC (40 pg/µL). Additionally, 40 µL of Proteinase K (20 mg/mL) was added for lysis and incubated at 70°C for 10 minutes. All DNA samples were resuspended in 50 µL of DNAse/RNAse-free water.

### Validatory satDNA TaqMan qPCR

A duplex qPCR was conducted for all clinical samples following the protocol previously published by Mayta et al. (40). Briefly, the PCR targets the satellite sequence of the nuclear genome of *T. cruzi* and IAC. The qPCR was performed according to published methods, using primers Cruzi 1 (5′–ASTCGGCTGATCGTTTTCGA–3′) and Cruzi 2 (5′–AATTCCTCCAAGCAGCGGATA–3′) to amplify a 166 bp fragment of nuclear satellite DNA. The probe cruzi 3 (5′–CACACACTGGACACCAA–3′) was labeled with 5′FAM (6–carboxyfluorescein) and 3′MGB (minor groove binder)(40).

### Alignment of HSP70 gene sequences

The *HSP70* sequence of *T. cruzi* Brazil A4 (TcBrA4_0077200, TriTryps DB) was used as input for a BLAST search against *T. cruzi* strains, *Leishmania*, and *T. rangeli* strains available in the TriTryps and National Center for Biotechnology Information databases. Representative sequences with the highest identity were retained for each strain. A total of 51 representative sequences were evaluated: 36 from *T. cruzi*, 11 from *Leishmania*, and four from *T. rangeli*. The *HSP70* sequences were analyzed using a neighbor-joining tree with Geneious Prime 2024.0.5 software. The parameters used were global alignment with free gaps, a cost matrix of 70% similarity (IUB) (5.0/−4.5), and the Tamura-Nei genetic distance model.

### LAMP*_TcHSP70_* primer design

Upon sequence alignment, primers were manually designed in regions with high coverage and identity for *T. cruzi* strains. Sequence alignment and primer annotation were completed using the Geneious Prime 2024.0.5 software. The primers were designed to maximize variants in *Leishmania* or *T. rangeli* homologous sequences. The likelihood of self-dimer and heterodimers was evaluated by the Oligoanalyzer IDT tool using default parameters.

### LAMP master mix and incubation

The master mix preparation was adapted from a protocol by Ordóñez et al. (33). The master mix was prepared in batches of 50–100 reactions in 23 µL and contained: 40 pmol FIP primer, 40 pmol BIP primer, 0.5 pmol F3 primer, 0.5 pmol B3 primer, 0.5 pmol LF primer, 0.5 pmol LB primer, 2.5 mM dNTPs (NEB, CN: N0447S), 0.8 mM MgSO₄ (NEB, CN: B1003S), 1 M Betaine (Sigma-Aldrich, CN: B0300-5VL), 1× Isothermal Amplification Buffer (20 mM Tris-HCl pH 8.8, NEB, CN: B0537S), and 8 U Bst Polymerase 2.0 WarmStart (NEB, CN: M0537L).

Two microliters of DNA template were added to the master mix to achieve a total volume of 25 µL. SYBR Green I dye (2 µL of a 1:10 dilution) (Invitrogen, CN: S-7567) was then added to the tube lid of the tube. By default, the reaction was incubated at 65°C for 60 minutes. After incubation, the vials were centrifuged to mix the dye with the amplified product.

### LAMP visualization

The amplified products were initially visualized by the eye using a black background. For quantitative analysis, the amplified products were imaged using an Azure Biosystems 200 gel imager with a 365 nm light source and a 595 nm emission filter. The mean fluorescence intensity was calculated using the ROI manager function of Fiji2 software.

Additionally, during standardization, the amplified products were also visualized on agarose gel. The products were pre-incubated at 80°C for 10 minutes to deactivate the enzyme and stop the reaction. Then, 3 µL of the amplified products were visualized on a 2% agarose gel (Invitrogen, CN: 16-500-500), pre-stained with 0.2 µg/ml ethidium bromide. The electrophoresis was performed at 100V for 35 minutes.

### Analytical LoD LAMP*_TcHSP70_*

DNA from seven *T. cruzi* strains (Dm28c_DTU-I, Sylvio_DTU-I, Perú_DTU-II, Y-DTU-II, TcIII_Col_DTU-III, VT-1_DTU-II, Tulahuen_DTU-VI, CL-DTU-VI) was purified and quantified using a Qubit fluorometer (Thermo Fisher, CN: Q32850). The DNA concentrations were normalized to 0.5 ng/µL, resulting in a total of 1 ng in 2 µL when added to the master mix. The DNA stocks were initially subjected to a broad 1:10 dilution. Two representative strains (Dm28c_DTU-I and Perú_DTU-II) were then further diluted in a series of 1:2 dilutions to refine the LoD. The analytical LoD was defined as the lowest DNA concentration that resulted in positive amplification.

To calculate the equivalent number of genome/parasite copies, we used the formula:


$$Number\;of\;copies=\frac{ng\;of\;DNA\;\left(LoD\right)*Avogadro^{\prime }s\;number}{genome\;size\left(bp\right)*\frac{1x10^{9}ng}{g}*\frac{660g}{mole\;of\;bp}}$$

The genome size for nine strains (G, Dm28c, Tc1161, Y, Esmeraldo, SO3-cl5, CL, CL14, and CL Brener) was obtained from Souza et al. (52). The LoD was fixed at 0.00125 ng. The estimated number of copies was then divided by the median gene dose for *HSP70* in *T. cruzi*, which is 88, as determined using the gene copy number tool of TriTrypDB (https://tritrypdb.org/tritrypdb/app/search/transcript/GenesByCopyNumber).

### Stability of LAMP-TcHSP70 across different incubation times

The LAMP-TcHSP70 reaction was incubated at 65°C for 30, 40, 50, 60, 80, and 120 minutes. Each incubation time was tested in at least four replicates. Reactions were seeded with 2 μL containing ~2 pg of *T. cruzi* (Dm28c strain) DNA as a positive control and 50 ng of human DNA as a negative control. For no-template controls, DNA was replaced with molecular-grade water. Immediately after incubation, samples were spun down, and the amplification was detected by eye and fluorescence.

### Stability of the complete LAMP*_TcHSP70_* master mix at 4°C

Twenty-four LAMP reactions were prepared and stored at 4°C. Four reactions were randomly selected and subjected to amplification at six time points (weeks 0, 1, 2, 4, 5, and 8 post-storage). At each time point, three reactions were seeded with 1 ng of *T. cruzi* DNA (Dm28c_DTU-I), and the remaining vial was loaded with 50 ng of human DNA (negative control). Immediately after incubation, samples were spun down to mix the SYBR Green, and fluorescence was measured using a 365 nm excitation light source and a 595 nm emission filter.

### Stability of the complete LAMP*_TcHSP70_* master mix at −20°C

Twenty-four LAMP reactions were prepared and stored at −20°C. Four reactions were randomly selected, thawed, and subjected to amplification at six time points (months 0, 1, 2, 4, 6, and 8 post-storage). At each time point, three vials were seeded with 1 ng of *T. cruzi* DNA (Dm28c_DTU-I), and the remaining vial was loaded with 50 ng of human DNA (negative control). Immediately after incubation, samples were spun down to mix the SYBR Green, and fluorescence was measured using a 365 nm excitation light source and a 595 nm emission filter.

### One-pot visual detection using Alt-R CRISPR-Cas12a

The Cas12a-LAMP one-pot detection system was adapted from Qin et al. (39). In a vial containing the previously described LAMP master mix, 2 µL of DNA template was added, followed by 35 µL of PCR-grade mineral oil. On top of the vial, 20 µL of the Cas12a reaction mix was added, which included 2× NEB Buffer r2.1 (NEB, CN: B6002S), 8 U RNAase Inhibitor (Thermo Fisher, CN: N8080119), and 0.125 µM gRNA (5′- /AlTR1/rUrArArUrUrUrCrUrArCrUrArArGrUrGrUrArGrArUrCrGrUrCrArArUrGrCrGrCrUrCrGrCrGrCrUrGrU/AlTR2/−3′), 0.125 µM Alt-R L.b. Cas12a (Cpf1) Ultra (IDT, CN: 430891851), and 0.4 µM ssDNA probe (/56-FAM/TTATTATT/3IABkFQ/). The reaction was incubated at 65°C for 40 minutes. After incubation, the reaction was briefly spun down for 10 seconds to mix the Cas12a reaction mix with the LAMP amplified products. The mixture was then incubated again at 37°C for 20 minutes. Products were imaged under LED blue light using an Azure Biosystems 200 imaging system.

### Comparison of CTAB-based and commercial kit DNA extraction for LAMP-PCR

Fresh EDTA-treated human blood was spiked with *T. cruzi* (1 × 10⁴ parasites/mL), followed by three serial 10-fold dilutions in blood. Two *T. cruzi* strains (Tulahuen-DTU VI and Y-DTU II) were tested across eight independent dilution series. DNA was extracted from 300 µL of whole blood using two methods: (i) the Roche High Pure PCR Template Preparation Kit (commercial method), following the manufacturer’s protocol, and (ii) a CTAB-based protocol (proposed alternative). For CTAB extraction, 500 µL of CTAB lysis buffer (2% CTAB, 100 mM Tris-HCl pH 8.0, 20  mM EDTA, 1.4 M NaCl) was added to blood, vortexed, and incubated at 65°C for 10 minutes, followed by 1 minutes on ice. Protein precipitation was performed with 500 µL of 5 M NaCl, followed by centrifugation (12,000 ×  *g*, 2  minutes). The supernatant was mixed with an equal volume of cold isopropanol, centrifuged (12,000 ×  *g*, 5  minutes), and the pellet was washed with 70% and 100% ethanol, dried at 70°C for 3 minutes, and resuspended in 50  µL of pre-heated nuclease-free water. DNA concentration was measured using a NanoDrop spectrophotometer.

LAMP_TcHSP70_ reactions were performed using 5 µL containing ~100 ng DNA, incubated at 65 °C for 1 h, and visually assessed with SYBR Green. Differences in DNA yield were assessed with a Wilcoxon rank-sum test. To evaluate how the extraction method affected LAMP-PCR performance, we fitted a logistic-regression model: Detection ~ Method  ×  log₁₀(Concentration). The CTAB-based method was used only for this comparison, while the High Pure PCR Template Preparation Kit was the default for all other blood samples.

### Diagnostic test performance metrics

The sensitivity, specificity, accuracy, and Cohen’s kappa agreement were estimated using the epiR (version 2.0.76) package in R, using a confidence level of 95%.

### Cost estimation for LAMP*_TcHSP70_***,** TaqMan SatDNA qPCR, and CTAB protocol

To calculate the total cost per reaction, the following categories were considered: reagents, disposable materials, personnel time, and permanent materials. All prices were based on costs in the United States. Annual depreciation for permanent materials was estimated using the formula: $Annual\;depreciation:\frac{Equipment\;price(\$)-salvage\;value(\$)}{useful\;life\;(years)}$

### *HSP70* amplicon sequencing

Parasites were isolated from patients in Santa Cruz, Bolivia. The parasites were grown in culture for 3–4 months before cryopreservation. Parasites were then extracted with QIAmp DNA Blood Mini Kit (QIAGEN, CN: 51104), and Illumina sequencing libraries were prepared with NEBNext Ultra II DNA Library Prep Kit (Illumina, E7645L) and barcoded with unique dual indexes for multiplexing. Libraries were sequenced in an Illumina NovaseqX with PE 150 bp reads. Reads aligned to *T. cruzi* were assembled using Spades v4.0.0 (53), with kmer lengths of 21,33,55,77,99, and 127 in paired-end mode. Heat shock protein sequences from these genomes were extracted from assemblies using a BLAST search against HSP70.

## Data Availability

All data produced in the present work are contained in the manuscript. A detailed protocol for the LAMP-PCR assay is available at dx.doi.org/10.17504/protocols.io.e6nvwbpqzvmk/v1. A detailed protocol for the CTAB DNA extraction protocol is available at dx.doi.org/10.17504/protocols.io.rm7vz9mnrgx1/v1. R code used to estimate the specificity, sensitivity, and Kappa agreement is available at http://rpubs.com/sgutierr/1262176.

## SUPPLEMENTAL MATERIAL

File S1 spectrum.00172-25-s0001.xlsx *T. cruzi* HSP70 distance and identity.

File S2 spectrum.00172-25-s0002.xlsx Primer sequences.

File S3 spectrum.00172-25-s0003.txt Representative alignment for HSP70 sequences.

File S4 spectrum.00172-25-s0004.xlsx Heterodimer evaluation.

File S5 spectrum.00172-25-s0005.xlsx Analytical limit of detection.

File S6 spectrum.00172-25-s0006.xlsx Comparison of the limit of detection between LAMP and qPCR.

File S7 spectrum.00172-25-s0007.xlsx Cost estimation for DNA extraction and LAMP-PCR.

File S8 spectrum.00172-25-s0008.xlsx LAMP extended incubation and long-term storage.

File S9 spectrum.00172-25-s0009.xlsx LAMP performance in clinical samples.

Supplemental Figures spectrum.00172-25-s0010.docx Extended LAMP stability and alternative DNA extraction method performance.

ASM does not own the copyrights to Supplemental Material that may be linked to, or accessed through, an article. The authors have granted ASM a non-exclusive, world-wide license to publish the Supplemental Material files. Please contact the corresponding author directly for reuse.
