# Supplementary material for: A specific, stable, and accessible LAMP assay targeting the HSP70 gene of Trypanosoma cruzi
Source: Microbiol Spectr. 2025 Oct 14;13(11):e00172-25. doi: 10.1128/spectrum.00172-25 (PMC12584694; doi:10.1128/spectrum.00172-25)
Supplement: Supplemental Figures — Extended LAMP stability and alternative DNA extraction method performance. [file spectrum.00172-25-s0010.docx]

**Supplementary Figure for manuscript:**

**A specific, stable, and accessible LAMP assay targeting the HSP70 gene of *Trypanosoma cruzi***

**Sneider Alexander Gutierrez Guarnizo^1^**, Beth Jessy Condori^2^, Luciana Basma^3^ , Shirley Equilia^4^, Edith Malaga^2^, Siena Defazio^1^, Emily Arteaga^4^, Jean Karla Velarde^4^_,_ Martín Obregón^2^, Anshule Takyar^1^, Carolina Duque^5^, Jill Hakim^6^, Freddy Tinajeros^4^, Robert H Gilman^1^, Natalie Bowman^7^, Monica R. Mugnier^6*^.

1. Department of International Health, Johns Hopkins University Bloomberg School of Public Health, Baltimore, MD, USA.

2. Infectious Diseases Research Laboratory, Department of Cellular and Molecular Sciences, Universidad Peruana Cayetano Heredia, Lima, Perú.

3. Universidad Católica Boliviana San Pablo. Santa Cruz, Bolivia.

4. Asociación Benéfica Prisma (PRISMA). Lima, Perú

5. Department of Pathology, Johns Hopkins School of Medicine, Baltimore, Maryland, USA.

6. W. Harry Feinstone Department of Molecular Microbiology and Immunology, Johns Hopkins Bloomberg School of Public Health, Baltimore, Maryland, USA.

7. Division of Infectious Diseases, School of Medicine, University of North Carolina at Chapel Hill, Chapel Hill, North Carolina.

*Address correspondence to Monica R. Mugnier: mmugnie1@jhu.edu

**
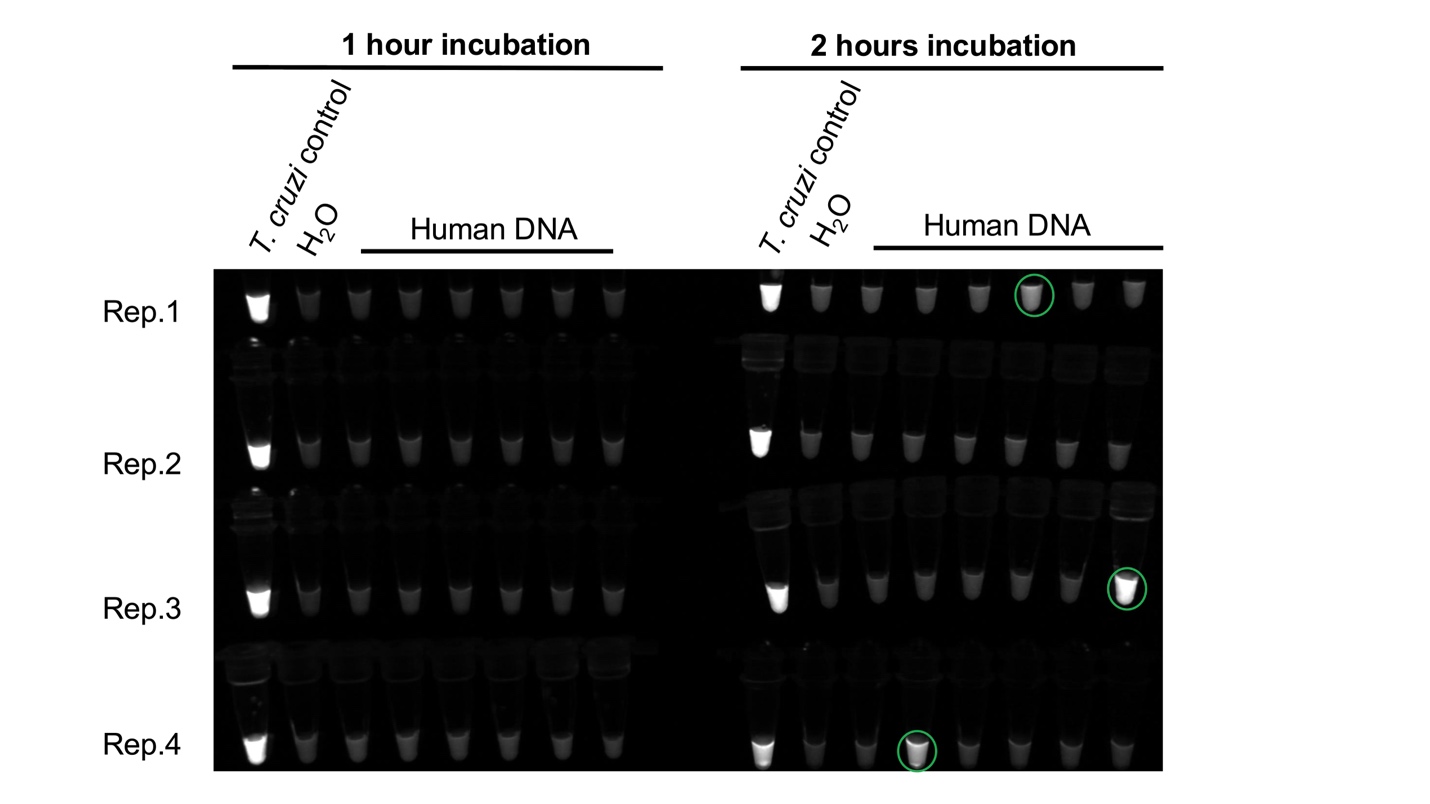
**

**Supplementary Figure 1. LAMP-PCR_TcHSP70_ shows stable performance after 1 hour of incubation but exhibits random nonspecific amplification after 2 hours.** Independent master mixes were seeded with 2 µL containing ~2 pg of T. cruzi (Dm28c strain) DNA as a positive control and 50 ng of human DNA (HEK cells) as a negative control. No template controls used molecular-grade water instead of DNA. Green circles highlight nonspecific amplification after 2 hours.

***
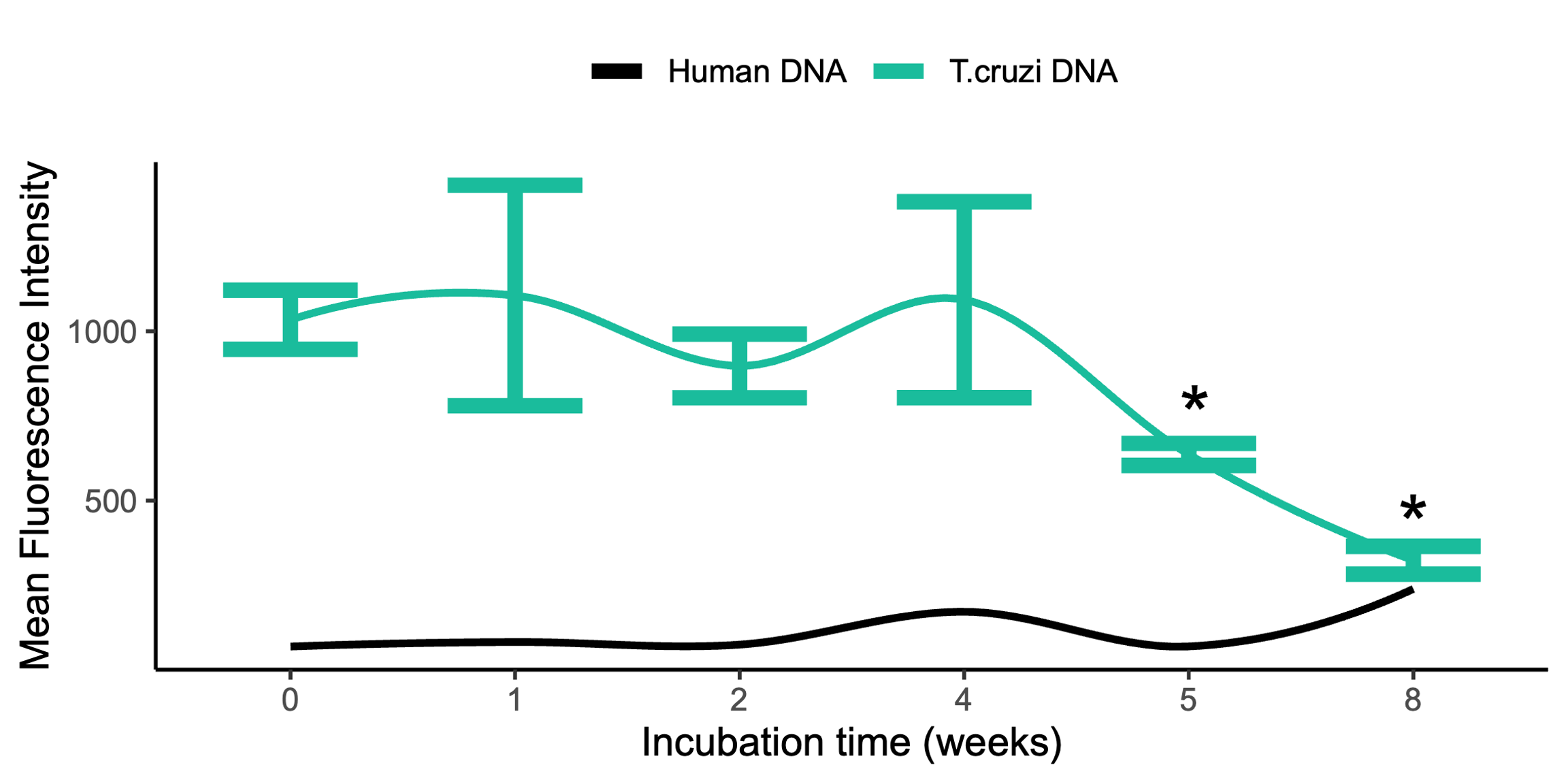
***

**Supplementary Figure 2.** **The LAMP_TcHsp70_ assay shows stability after storage at 4 °C for up to 4 weeks.** The LAMP_TcHsp70_ master mix was stored at 4 °C and used for amplification at weeks 0, 1, 2, 4, 5, and 8. Reactions were seeded with 1 ng of T. cruzi DNA or 50 ng of human DNA (negative control). A repeated measures ANOVA revealed a significant effect of storage time on fluorescence intensity (F = 8.499, p = 0.015). Post hoc pairwise comparisons were performed using paired t-tests; asterisks indicate statistically significant differences (p ≤ 0.05).


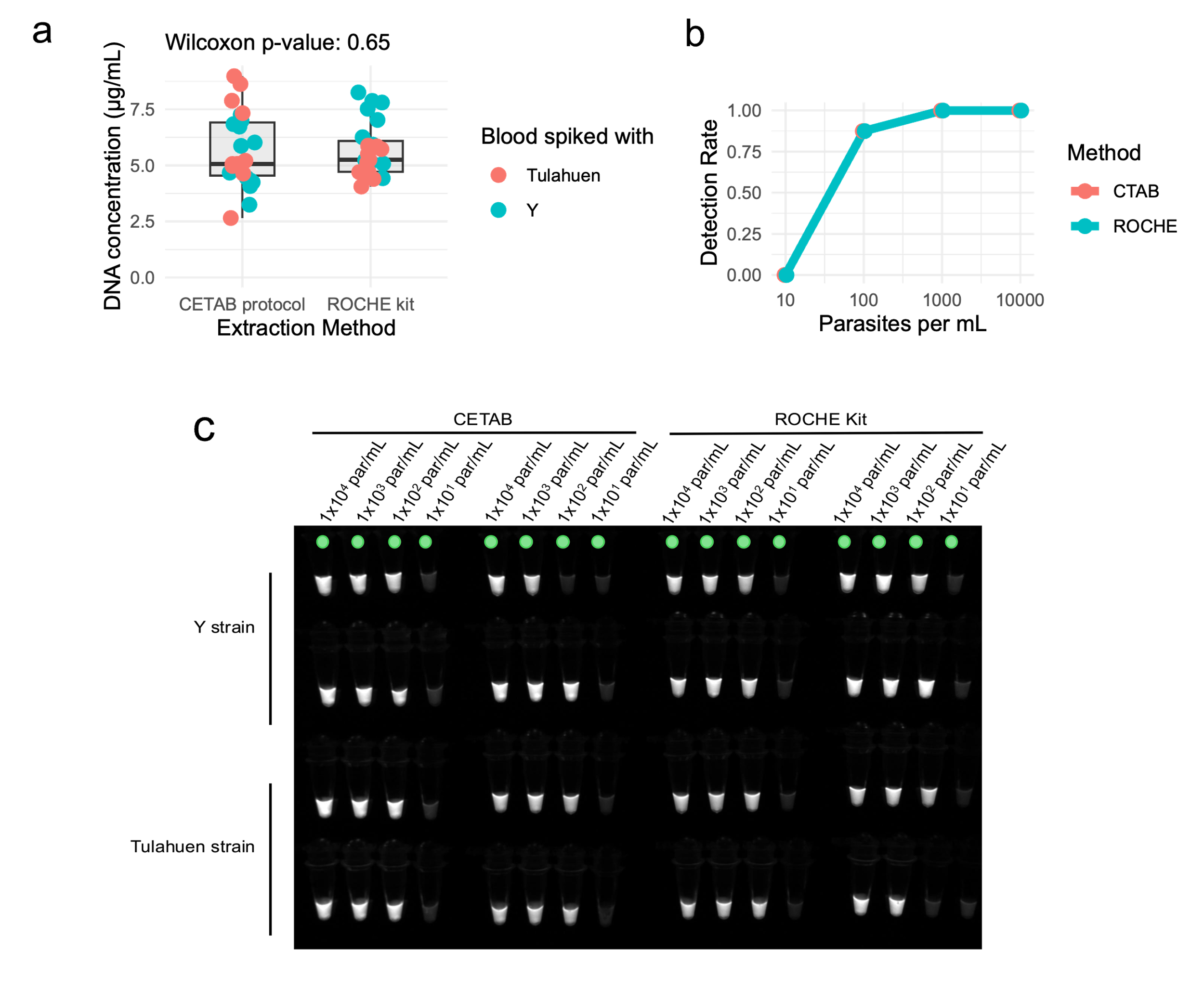


**Supplementary Figure 3. LAMP-PCR shows comparable amplification when using either blood DNA extracted by CTAB or the Roche High Pure PCR Template Preparation Kit.**
LAMP-PCR was performed on DNA extracted from human blood samples spiked with T. cruzi (1×10⁴ parasites/mL), followed by three serial 10-fold dilutions. Two DNA extraction protocols were compared: a CTAB-based method (alternative) and the Roche High Pure PCR Template Preparation Kit (reference). Two independent T. cruzi strains were tested (Tulahuen-DTU VI and Y-DTU II).
**a**, DNA concentration (μg/mL of whole blood) quantified by NanoDrop for each method. Medians were compared using the Wilcoxon rank-sum test (W = 265.5, P = 0.6501), showing no significant difference in DNA yield.
**b**, Detection rate of T. cruzi by LAMP-PCR for each DNA extraction method per parasite concentration in whole blood sample. Logistic regression analysis (Detection ~ Method × log₁₀[Concentration]) revealed no significant effect of extraction method, DNA concentration, or their interaction on the detection probability (P > 0.99 for all terms), indicating similar performance across methods.
**c**, Representative SYBR Green fluorescence results of LAMP-PCR. Non-visible signal indicates a negative result.
